# Supplementary material for: Environmental pathogen surveillance in cities without universal piped wastewater infrastructure
Source: PLOS Glob Public Health. 2026 Apr 10;6(4):e0004994. doi: 10.1371/journal.pgph.0004994 (PMC13068267; doi:10.1371/journal.pgph.0004994)
Supplement: S1 Fig — (PDF) [file pgph.0004994.s001.pdf]

S1 Fig. Detailed Map

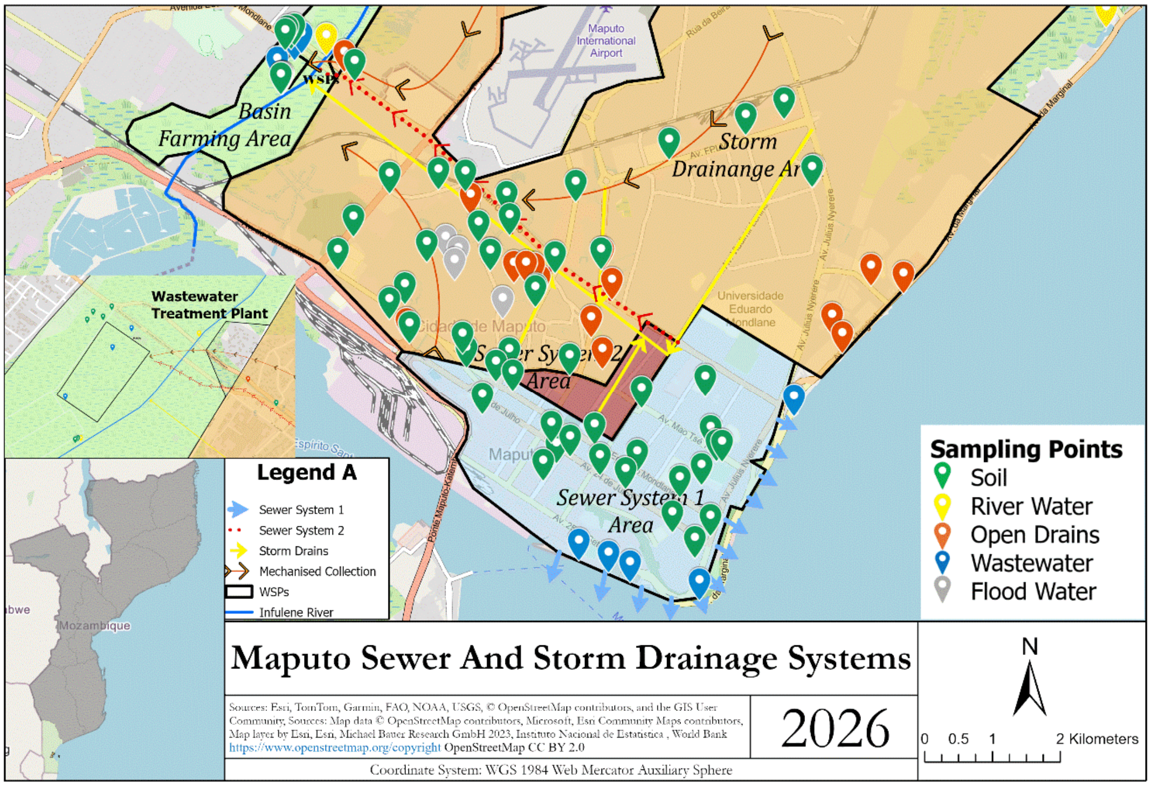

Map of sampling locations and sewer and storm drainage systems in Maputo, Mozambique.

Sampling locations are shown for soil (green), river water (yellow), open drains (orange), wastewater (blue), and floodwater (gray). Sewer System 1 and Sewer System 2, storm drainage networks, and mechanized fecal sludge collection routes are indicated. Waste stabilization ponds (WSPs) and the Infulene River are also shown.

Basemap data are derived from OpenStreetMap contributors (https://www.openstreetmap.org/copyright) and were accessed via Esri. OpenStreetMap data are licensed under the Open Database License (ODbL). The map was created by the authors using ArcGIS.

Coordinate system: WGS 1984 Web Mercator Auxiliary Sphere.
