# Supplementary material for: Environmental pathogen surveillance in cities without universal piped wastewater infrastructure
Source: PLOS Glob Public Health. 2026 Apr 10;6(4):e0004994. doi: 10.1371/journal.pgph.0004994 (PMC13068267; doi:10.1371/journal.pgph.0004994)
Supplement: S2 Fig — (PDF) [file pgph.0004994.s002.pdf]

S2 Fig. Maputo SFD

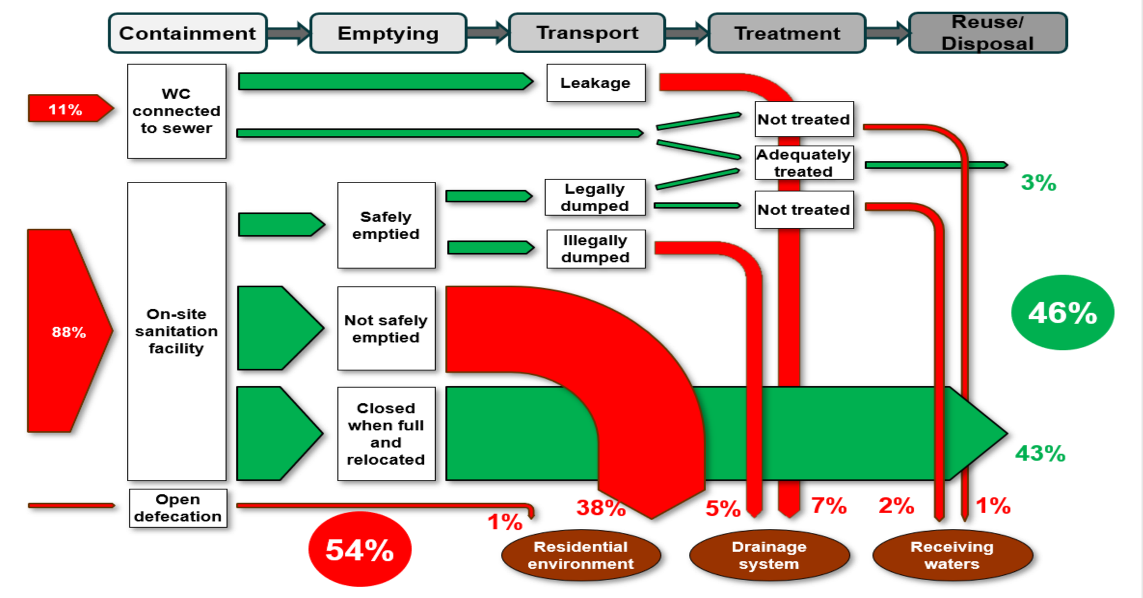

The excreta flow diagram (SFD) for Maputo was developed by World Bank staff in Maputo in 2013.[1] The SFD begins on the left with human excreta, which may enter into a sewage system, onsite sanitation system, or be openly defecated into the environment. Flows move toward the right, where they can be safely managed, or to the bottom, indicating unsafe management of excreta. Matrices covered by the SFD include wastewater, which in Maputo includes influent to the wastewater treatment plant (WWTP) as well as direct discharge to Maputo Bay via outfalls. Wastewater that reaches the WWTP is treated (though not very effectively) and discharged as effluent to the Infulene River. In Maputo, wastewater and fecal sludge may contaminate open drains which may drain into surface water or Maputo Bay. Child feces in diapers are often discarded as solid waste. However, many individuals make their living by sorting recyclables and frequently empty solid waste bins to aid in sorting, causing fecal contamination in the nearby soil (see Figure S4 showing diapers on the ground next to a waste bin). While not directly present in the SFD, we choose to include soil adjacent to public waste receptacles to capture this citywide fecal contamination. Due to clogged and inadequate drainage infrastructure, certain areas of Maputo are prone to pooling of stormwater (i.e., localized flooding) during the rainy season. Stormwater may mix with fecal wastes from onsite sanitation systems or existing contamination in open drains and soils. We choose to include stormwater as a point of comparison despite not being represented on the SFD.

The city experienced no major changes in public domain infrastructure from the creation of the SFD until our study, though maintenance to the WWTP and sewers did occur after the study from 2023-2025.[2,3] It estimated that 11% of the population was connected to a sewer, 88% used onsite sanitation, and 1% practiced open defecation. It was estimated that 25% of wastewater arrived at the WWTP and treatment was 50% effective. For onsite sanitation, 20% of waste was estimated to be safely covered and abandoned, 20% hygienically emptied using mechanized equipment, and the remaining 60% unhygienically emptied using manual equipment. All the unhygienically emptied waste and 25% of the hygienically emptied waste was estimated to be

dumped illegally. Finally, it was estimated that treatment of the onsite waste taken to the WWTP was 50% effective.
