# Supplementary material for: Environmental pathogen surveillance in cities without universal piped wastewater infrastructure
Source: PLOS Glob Public Health. 2026 Apr 10;6(4):e0004994. doi: 10.1371/journal.pgph.0004994 (PMC13068267; doi:10.1371/journal.pgph.0004994)

S3 Fig. BMFS Sample Collection

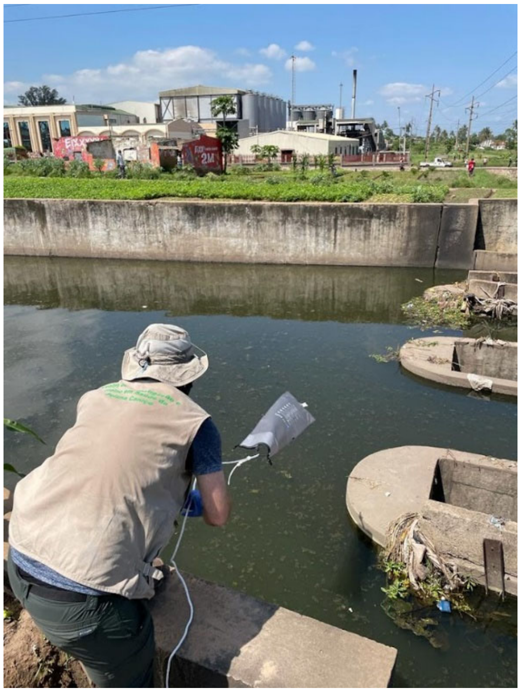

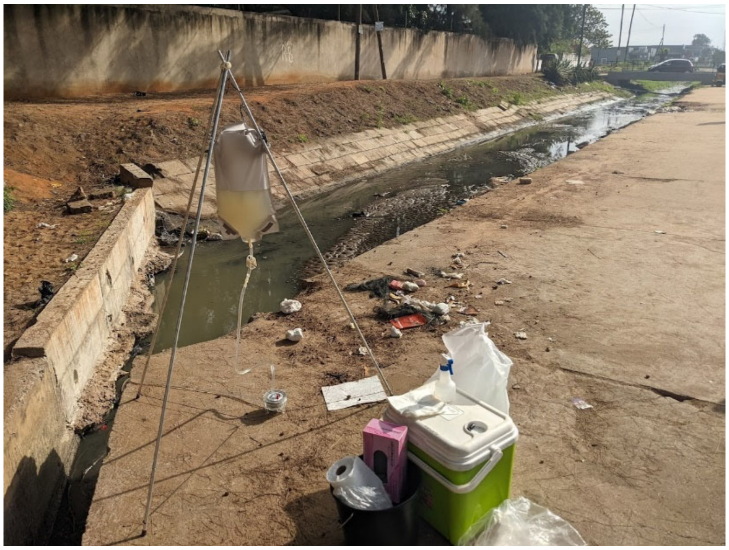

Open drains (above), Infulene river (bottom left), and a wastewater outfall into Maputo Bay (bottom right)

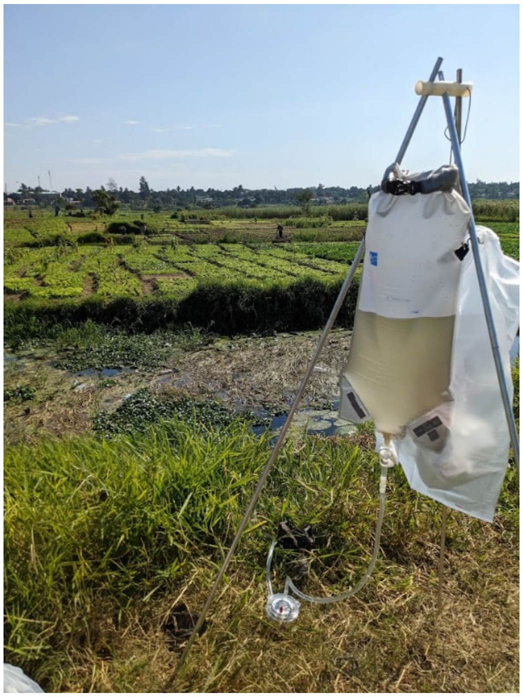

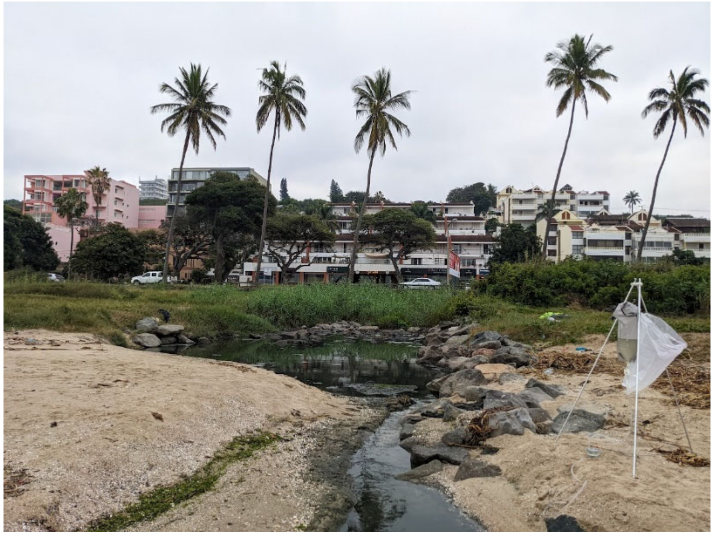

Supplement: S3 Fig — (PDF) [file pgph.0004994.s003.pdf]
