# Supplementary material for: Environmental pathogen surveillance in cities without universal piped wastewater infrastructure
Source: PLOS Glob Public Health. 2026 Apr 10;6(4):e0004994. doi: 10.1371/journal.pgph.0004994 (PMC13068267; doi:10.1371/journal.pgph.0004994)
Supplement: S4 Fig — (PDF) [file pgph.0004994.s004.pdf]

S4 Fig. Fecal sludge collection

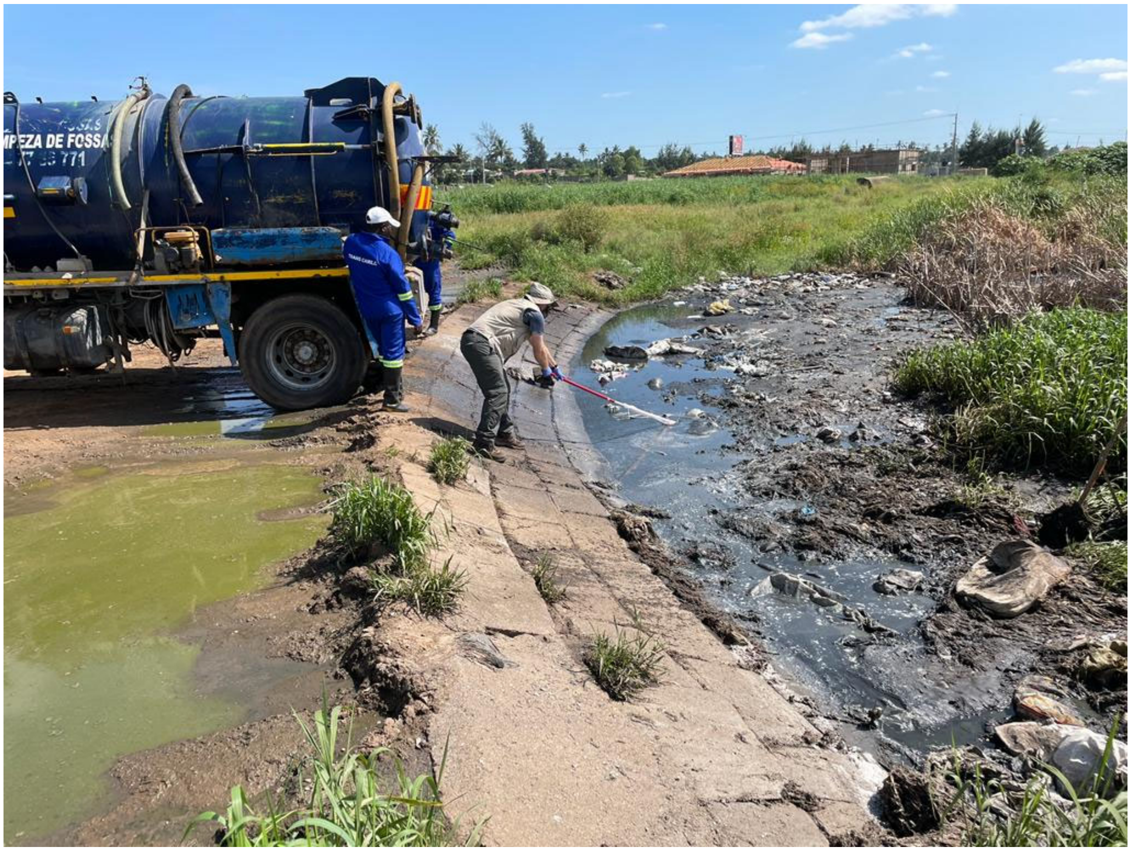

Note: Fecal sludge samples were collected using a sludge nabber at the point of discharge into the anaerobic pond at the wastewater treatment plant
