# Supplementary material for: Environmental pathogen surveillance in cities without universal piped wastewater infrastructure
Source: PLOS Glob Public Health. 2026 Apr 10;6(4):e0004994. doi: 10.1371/journal.pgph.0004994 (PMC13068267; doi:10.1371/journal.pgph.0004994)
Supplement: S1 Table — (PDF) [file pgph.0004994.s006.pdf]

S1 Table. Assays

#	Organism Name	Gene Name	Oligonucleotide Primer or Probe Sequence (5' – 3')	Ref
**1**	adenovirus 40-41	Fiber gene	Fwd: AACTTTCTCTCTTAATAGACGCC<br>Rev: AGGGGGCTAGAAAACAAAA<br>Probe: CTGACACGGGCACTCT	[12]
**2**	astrovirus	Capsid	Fwd: CAGTTGCTTGCTGCGTTCA<br>Rev: CTTGCTAGCCATCACACTTCT<br>Probe: CACAGAAGAGCAACTCCATCGC	[12]
**3**	norovirus GI	ORF1-2	Fwd: CGCTGGATGCGATTCCATGA<br>Rev: CTTAGACGCCATCATCATTTAC<br>Probe: TGGACAGGAGATCGC	[12]
**4**	norovirus GII	ORF1-2	Fwd: CAAGAACCTATGTTTAGATGGATGAG<br>Rev: TCGACGCCATCTTCATTCACA<br>Probe: TGGGAGGGCGATCGCAATCT	[12]
**5**	rotavirus	NSP3	Fwd: ACCATCTTCACGTAACCCTCTATGAG<br>Rev: GGTCACATAACGCCCCTATAGC<br>Probe: AGTTAAAAGCTAACACTGTCAAA	[12]
**6**	sapovirus	RdRp	Fwd 1: GACCAGGCTCTCGCCACCTAC<br>Fwd 2: TTGGCCCTCGCCACCTAC<br>Rev: CCCTCCATTTCAAACACTA<br>Probe: CCGCCTATGAACCA	[12]
**7**	EAEC	aaiC	Fwd: ATTGTCCTCAGGCATTTCAC<br>Rev: ACGACACCCCTGATAAACAA<br>Probe: TAGTGCATACTCATCATTTAAG	[12]
**8**	EAEC	aatA	Fwd: CTGGCGAAAGACTGTATCAT<br>Rev: TTTTGCTTCATAAGCCGATAGA<br>Probe: TGGTTCTCATCTATTACAGACAGC	[12]
**9**	STEC	*stx1*	Fwd: ACTTCTCGACTGCAAAGACGTATG<br>Rev: ACAAATTATCCCCTGWGCCACTATC	[12]

			Probe: CTCTGCAATAGGTACTCCA	
**10**	STEC	*stx2*	Fwd: CCACATCGGTGTCTGTTATTAACC<br>Rev: GGTCAAAACGCGCCTGATAG<br>Probe: TTGCTGTGGATATACGAGG	[12]
**11**	*E. coli* O157:H7	*rfbE*	Fwd: TTTCACACTTATTGGATGGTCTCAA<br>Rev: CGATGAGTTTATCTGCAAGGTGAT<br>Probe: CTCTCTTTCCTCTGCGGTCCT	[12]
**12**	EPEC	eae	Fwd: CATTGATCAGGATTTTTCTGGTGATA<br>Rev: CTCATGCGGAAATAGCCGTTA<br>Probe: ATACTGGCGAGACTATTTCAA	[12]
**13**	EPEC	bfpA	Fwd: TGGTGCTTGCGCTTGCT<br>Rev: CGTTGCGCTCATTACTTCTG<br>Probe: CAGTCTGCGTCTGATTCCAA	[12]
**14**	ETEC	LT	Fwd: TTCCCACCGGATCACCAA<br>Rev: CAACCTTGTGGTGCATGATGA<br>Probe: CTTGGAGAGAAGAACCCT	[12]
**15**	ETEC	STh	Fwd: GCTAAACCAGCAGGGTCTTCAAAA<br>Rev: CCCGGTACAAGCAGGATTACAACA<br>Probe: TGGTCCTGAAAGCATGAA	[12]
**16**	ETEC	STp	Fwd: TGAATCACTTGACTCTTCAAAA<br>Rev: GGCAGGATTACAACAAAGTT<br>Probe: TGAACAACACATTTTACTGCT	[12]
**17**	*Shigella*/EIEC	*ipaH*	Fwd: CCTTTTCCGCGTTCCTTGA<br>Rev: CGGAATCCGGAGGTATTGC<br>Probe: CGCCTTTCCGATACCGTCTCTGCA	[12]
**18**	*Campylobacter jejuni/coli*	cadF	Fwd: CTGCTAAACCATAGAAATAAAATTTCTCAC<br>Rev: CTTTGAAGGTAATTTAGATATGGATAATCG<br>Probe: CATTTTGACGATTTTTGGCTTGA	[12]
**19**	*Salmonella enterica*	*ttr*	Fwd: CTCACCAGGAGATTACAACATGG<br>Rev: AGCTCAGACCAAAAGTGACCATC<br>Probe: CACCGACGGCGAGACCGACTTT	[12]

**20**	*Vibrio cholerae*	*hylA*	Fwd: ATCGTCAGTTTGGAGCCAGT<br>Rev: TCGATGCGTTAAACACGAAG<br>Probe: ACCGATGCGATTGCCCAA	[12]
**21**	*Clostridium difficile*	tcdB	Fwd: GGTATTACCTAATGCTCCAAATAG<br>Rev: TTTGTGCCATCATTTTCTAAGC<br>Probe: CCTGGTGTCCATCCTGTTTC	[12]
**22**	*Aeromonas* spp.	Aerolysin	Fwd: TTCGTTACCAGTGGGACAAG<br>Rev: CCGGCAAACTGGCTCTCG<br>Probe: CAGTTCCAGTCCCACCACTT	[12]
**23**	*Helicobacter pylori*	ureC	Fwd: GACACCAGAAAAAGCGGCTA<br>Rev: AGCGCATGTCTTCGGTTAAA<br>Probe: TCACTAAAGCGTTTTCTACC	[12]
**24**	*Cryptosporidium* spp.	18S rRNA	Fwd: GGGTTGTATTTATTAGATAAAGAACCA<br>Rev: AGGCCAATACCCTACCGTCT<br>Probe: TGACATATCATTCAAGTTTCTGAC	[12]
**25**	*Giardia* spp.	18S rRNA	Fwd: GACGGCTCAGGACAACGGTT<br>Rev: TTGCCAGCGGTGTCCG<br>Probe: CCCGCGGCGGTCCCTGCTAG	[12]
**26**	*Entamoeba histolytica*	18S rRNA	Fwd: ATTGTCGTGGCATCCTAACTCA<br>Rev: GCGGACGGCTCATTATAACA<br>Probe: TCATTGAATGAATTGGCCATTT	[12]
**27**	*Ascaris lumbricoides*	*ITS1*	Fwd: GCCACATAGTAAATTGCACACAAAT<br>Rev: GCCTTTCTAACAAGCCCAACAT<br>Probe: TTGGCGGACAATTGCATGCGAT	[12]
**28**	*Trichuris trichiura*	18S rRNA	Fwd: TTGAAACGACTTGCTCATCAACTT<br>Rev: CTGATTCTCCGTTAACCGTTGTC<br>Probe: CGATGGTACGCTACGTGCTTACCATGG	[12]
**29**	*Ancylostoma duodenale*	*ITS2*	Fwd: GAATGACAGCAAACTCGTTGTTG<br>Rev: ATACTAGCCACTGCCGAAACGT<br>Probe: ATCGTTTACCGACTTTAG	[12]

**30**	*Necator americanus*	*ITS2*	Fwd: CTGTTTGTCGAACGGTACTTGC<br>Rev: ATAACAGCGTGCACATGTTGC<br>Probe: CTGTACTACGCATTGTATAC	[12]
**31**	BRSV	BRSV	Fwd: GCAATGCTGCAGGACTAGGTATAAT<br>Rev: ACACTGTAATTGATGACCCCATTCT<br>Probe: ACCAAGACTTGTATGATGCTGCCAAAGCA	[19]
**32**	BHV	BHV	Fwd: GAGCAAAGCCCCGCCGAAGGA<br>Rev: TACGAACAGCAGCACGGGCGG<br>Probe: GAACCTGCCCACGCGCTGAAAC	[20]
**33**	pan-16S	16S	Fwd: ATGGYTGTCGTCAGCT<br>Rev: ACGGGCGGTGTGTAC<br>Probe: CAACGAGCGCAACCC	[21]
**34**	*Mycobacterium tuberculosis*	IS6110	Fwd: GGGTAGCAGACCTCACCTATG<br>Rev: AGCGTAGGCGTCGGTGA<br>Probe: TCGCCTACGTGGCCTTT	[22]
**35**	Toxocara spp.	*cox1*	Fwd: AAAATAGCCAAATCCACACTACTACCA<br>Rev: GGTGTGGGACTAGTTGAACTGTGTA<br>Probe: CCCCATAGTCCTCAAAG	[5]
**36**	Hs99999901_s1	18s	Thermofisher Internal Control	
**37**	SARS-CoV-2	N1	Fwd: GACCCCAAAATCAGCGAAAT<br>Rev: TCTGGTTACTGCCAGTTGAATCTG<br>Probe: ACCCCGCATTACGTTTGGTGGACC	[6]
**38**	avian 16s	Av4143	Fwd: TGCAAGTCGAACGAGGATTTCT<br>Rev: TCACCTTGGTAGGCCGTTACC<br>Probe: AGGTGGTTTTGCTATCGCTTT	[7]
**39**	HIV proviral DNA	capsid p24	Fwd: TGGGTAAAAGTAGTAGAAGAGAAGGCTTT<br>Rev: GCTATGTCACTTCCCCTTGGTTCT<br>Probe: TCAGCATTATCAGAAGGAG	[8]
**40**	*Plasmodium* spp.		Fwd: GTTAAGGGAGTGAAGACGATCAGA<br>Rev: AACCCAAAGACTTTGATTTCTCATAA	[23]

			Probe: CCGTCGTAATCTTAACCATAAACTATGCCGACT	
**41**	Leptospira spp.	*lipL32*	Fwd: AAGCATTACCGCTTGTGGTG<br>Rev: GAACTCCCATTTCAGCGATT<br>Probe: AAAGCCAGGACAAGCGCCG	[10]
**42**	poultry mtDNA	*cytb*	Fwd: AAATCCCACCCCCTACTAAAAATAAT<br>Rev: CAGATGAAGAAGAATGAGGCG<br>Probe: ACAACTCCCTAATCGACCT	[11]
**43**	EAEC2	aggR	Fwd: GCAATCAGATTAAGCAGCGATACA<br>Rev: TTCGGACAACTGCAAGCATC<br>Probe: AAGACGCCTAAAGGATGCCC	[12]
**44**	hCYTB484 (human mtDNA)	cytochrome *b*	Fwd: CAATGAATCTGAGGAGGCTAC<br>Rev: CGTGCAAGAATAGGAGGTG<br>Probe: ACCCTCACACGATTCTTTACCTTTCACT	[13]
**45**	intI1	intI1	Fwd: GCCTTGATGTTACCCGAGAG<br>Rev: GATCGGTCGAATGCGTGT<br>Probe: ATTCCTCGTGGTTCTGGGTTTT	[24]
**46**	zika	ZIKV	Fwd: CCGCTGCCCAACACAAG<br>Rev: CCACTAACGTTCTTTTGCAGACAT<br>Probe: AGCCTACCTTGACAAGCAGTCAGACACTCAA	[25]
**47**	canine mtDNA	NADH subunit 5 marker	Fwd: GGCATGCCTTTCCTTACAGGATTC<br>Rev: GGGATGTGGCAACGAGTGTAATTATG<br>Probe: TCATCGAGTCCGCTAACACGTCGAAT	[26]
**48**	hepatitis G	hepG	Fwd: CGGCCAAAAGGTGGTGGATG<br>Rev: CGACGAGCCTGACGTCGGG<br>Probe: AGGTCCCTCTGGCGCTTGTGGCGAG	[12]

Cycling conditions with a 1C/s ramp rate between all steps: 45°C for 20 minutes, 95°C for 10 minutes, then 50 cycles of 95°C for 15 seconds and 60°C for 1 minute.

References

(1) Hawkins, P.; Muxímpua, O. Developing Business Models for Fecal Sludge Management in Maputo. *Water and Sanitation Program: Report* **2015**, No. June.

(2) van Esch, M. S.; van Ramshorst, J. G. V. *The Sewer System of Urban Maputo*; 2014.

(3) *Sanitation and Drainage Master Plan for the Greater Maputo Metropolitan Area*; Maputo, 2015.

(4) Zhou, N. A.; Fagnant-Sperati, C. S.; Komen, E.; Mwangi, B.; Mukubi, J.; Nyangao, J.; Hassan, J.; Chepkurui, A.; Maina, C.; van Zyl, W. B.; Matsapola, P. N.; Wolfaardt, M.; Ngwana, F. B.; Jeffries-Miles, S.; Coulliette-Salmond, A.; Peñaranda, S.; Shirai, J. H.; Kossik, A. L.; Beck, N. K.; Wilmouth, R.; Boyle, D. S.; Burns, C. C.; Taylor, M. B.; Borus, P.; Meschke, J. S. Feasibility of the Bag-Mediated Filtration System for Environmental Surveillance of Poliovirus in Kenya. *Food Environ Virol* **2020**, *12* (1), 35–47. https://doi.org/10.1007/s12560-019-09412-1.

(5) Fagnant, C. S.; Sánchez-Gonzalez, L. M.; Zhou, N. A.; Falman, J. C.; Eisenstein, M.; Guelig, D.; Ockerman, B.; Guan, Y.; Kossik, A. L.; Linden, Y. S.; Beck, N. K.; Wilmouth, R.; Komen, E.; Mwangi, B.; Nyangao, J.; Shirai, J. H.; Novosselov, I.; Borus, P.; Boyle, D. S.; Meschke, J. S. Improvement of the Bag-Mediated Filtration System for Sampling Wastewater and Wastewater-Impacted Waters. *Food Environ Virol* **2018**, *10* (1), 72–82. https://doi.org/10.1007/s12560-017-9311-7.

(6) Zhou, N. A.; Fagnant-Sperati, C. S.; Shirai, J. H.; Sharif, S.; Zaidi, S. Z.; Rehman, L.; Hussain, J.; Agha, R.; Shaukat, S.; Alam, M.; Khurshid, A.; Mujtaba, G.; Salman, M.; Safdar, R. M.; Mahamud, A.; Ahmed, J.; Khan, S.; Kossik, A. L.; Beck, N. K.; Matrajt, G.; Asghar, H.; Bandyopadhyay, A. S.; Boyle, D. S.; Meschke, J. S. Evaluation of the Bag-Mediated Filtration System as a Novel Tool for Poliovirus Environmental Surveillance: Results from a Comparative Field Study in Pakistan. *PLoS One* **2018**, *13* (7), e0200551. https://doi.org/10.1371/journal.pone.0200551.

(7) Capone, D. Pathogens in the Public Domain https://osf.io/h5epg/ (accessed Apr 29, 2024). https://doi.org/10.17605/OSF.IO/H5EPG.

(8) Fagnant, C. S.; Toles, M.; Zhou, N. A.; Powell, J.; Adolphsen, J.; Guan, Y.; Ockerman, B.; Shirai, J. H.; Boyle, D. S.; Novosselov, I.; Meschke, J. S. Development of an Elution Device for ViroCap Virus Filters. *Environ Monit Assess* **2017**, *189* (11), 574. https://doi.org/10.1007/s10661-017-6258-y.

(9) Capone, D.; Berendes, D.; Cumming, O.; Knee, J.; Nalá, R.; Risk, B. B.; Stauber, C.; Zhu, K.; Brown, J. Analysis of Fecal Sludges Reveals Common Enteric Pathogens in Urban Maputo, Mozambique. *Environ Sci Technol Lett* **2020**, *7* (12), 889–895. https://doi.org/10.1021/acs.estlett.0c00610.

(10) Jalilian, J.; Moghaddam, S. S.; Tagizadeh, Y. Accelerating Soil Moisture Determination with Microwave Oven. *J. of Chinese Soil and Water Conserv* **2017**, *48* (2), 101–103.

(11) Borchardt, M. A.; Boehm, A. B.; Salit, M.; Spencer, S. K.; Wigginton, K. R.; Noble, R. T. The Environmental Microbiology Minimum Information (EMMI) Guidelines: QPCR and DPCR Quality and Reporting for Environmental Microbiology. *Environ Sci Technol* **2021**, *55* (15), 10210–10223. https://doi.org/10.1021/acs.est.1c01767.

(12) Liu, J.; Gratz, J.; Amour, C.; Nshama, R.; Walongo, T.; Maro, A.; Mduma, E.; Platts-Mills, J.; Boisen, N.; Nataro, J.; Haverstick, D. M.; Kabir, F.; Lertsethtakarn, P.; Silapong, S.; Jeamwattanalert, P.; Bodhidatta, L.; Mason, C.; Begum, S.; Haque, R.; Praharaj, I.; Kang, G.; Houpt, E. R. Optimization of Quantitative PCR Methods for Enteropathogen Detection. *PLoS One* **2016**, *11* (6), e0158199. https://doi.org/10.1371/journal.pone.0158199.

(13) Zhu, K.; Suttner, B.; Pickering, A.; Konstantinidis, K. T.; Brown, J. A Novel Droplet Digital PCR Human MtDNA Assay for Fecal Source Tracking. *Water Res* **2020**, *183*, 116085. https://doi.org/10.1016/j.watres.2020.116085.

(14) Caldwell, J. M.; Levine, J. F. Domestic Wastewater Influent Profiling Using Mitochondrial Real-Time PCR for Source Tracking Animal Contamination. *J Microbiol Methods* **2009**, *77* (1), 17–22. https://doi.org/10.1016/j.mimet.2008.11.007.

(15) Schiaffino, F.; Pisanic, N.; Colston, J. M.; Rengifo, D.; Paredes Olortegui, M.; Shapiama, V.; Peñataro Yori, P.; Heaney, C. D.; Davis, M. F.; Kosek, M. N. Validation of Microbial Source Tracking Markers for the Attribution of Fecal Contamination in Indoor-Household Environments of the Peruvian Amazon. *Science of The Total Environment* **2020**, *743*, 140531. https://doi.org/10.1016/j.scitotenv.2020.140531.

(16) Knapp, J.; Umhang, G.; Poulle, M.-L.; Millon, L. Development of a Real-Time PCR for a Sensitive One-Step Coprodiagnosis Allowing Both the Identification of Carnivore Feces and the Detection of Toxocara Spp. and Echinococcus Multilocularis. *Appl Environ Microbiol* **2016**, *82* (10), 2950–2958. https://doi.org/10.1128/AEM.03467-15.

(17) Gillings, M. R.; Gaze, W. H.; Pruden, A.; Smalla, K.; Tiedje, J. M.; Zhu, Y.-G. Using the Class 1 Integron-Integrase Gene as a Proxy for Anthropogenic Pollution. *ISME J* **2015**, *9* (6), 1269–1279. https://doi.org/10.1038/ismej.2014.226.

(18) Klymus, K. E.; Merkes, C. M.; Allison, M. J.; Goldberg, C. S.; Helbing, C. C.; Hunter, M. E.; Jackson, C. A.; Lance, R. F.; Mangan, A. M.; Monroe, E. M.; Piaggio, A. J.; Stokdyk, J. P.; Wilson, C. C.; Richter, C. A. Reporting the Limits of Detection and Quantification for Environmental DNA Assays. *Environmental DNA* **2020**, *2* (3), 271–282. https://doi.org/10.1002/edn3.29.

(19) Boxus, M.; Letellier, C.; Kerkhofs, P. Real Time RT-PCR for the Detection and Quantitation of Bovine Respiratory Syncytial Virus. *J Virol Methods* **2005**, *125* (2), 125–130. https://doi.org/10.1016/j.jviromet.2005.01.008.

(20) Wang, J.; O'Keefe, J.; Orr, D.; Loth, L.; Banks, M.; Wakeley, P.; West, D.; Card, R.; Ibata, G.; Van Maanen, K.; Thoren, P.; Isaksson, M.; Kerkhofs, P. Validation of a Real-Time PCR Assay for

the Detection of Bovine Herpesvirus 1 in Bovine Semen. *J Virol Methods* **2007**, *144* (1–2), 103–108. https://doi.org/10.1016/j.jviromet.2007.04.002.

(21) Ritalahti, K. M.; Amos, B. K.; Sung, Y.; Wu, Q.; Koenigsberg, S. S.; Löffler, F. E. Quantitative PCR Targeting 16S RRNA and Reductive Dehalogenase Genes Simultaneously Monitors Multiple Dehalococcoides Strains. *Appl Environ Microbiol* **2006**, *72* (4), 2765–2774. https://doi.org/10.1128/AEM.72.4.2765-2774.2006/ASSET/DC08ABD9-512C-48FF-ACC7-928ADF4672D1/ASSETS/GRAPHIC/ZAM0040666430004.JPEG.

(22) Liu, J.; Gratz, J.; Amour, C.; Nshama, R.; Walongo, T.; Maro, A.; Mduma, E.; Platts-Mills, J.; Boisen, N.; Nataro, J.; Haverstick, D. M.; Kabir, F.; Lertsethtakarn, P.; Silapong, S.; Jeamwattanalert, P.; Bodhidatta, L.; Mason, C.; Begum, S.; Haque, R.; Praharaj, I.; Kang, G.; Houpt, E. R. Optimization of Quantitative PCR Methods for Enteropathogen Detection. *PLoS One* **2016**, *11* (6), e0158199. https://doi.org/10.1371/JOURNAL.PONE.0158199.

(23) Lee, P. C.; Chong, E. T. J.; Anderios, F.; AL Lim, Y.; Chew, C. H.; Chua, K. H. Molecular Detection of Human Plasmodium Species in Sabah Using PlasmoNex^{TM} Multiplex PCR and Hydrolysis Probes Real-Time PCR. *Malar J* **2015**, *14* (1), 28. https://doi.org/10.1186/s12936-015-0542-5.

(24) Barraud, O.; Baclet, M. C.; Denis, F.; Ploy, M. C. Quantitative Multiplex Real-Time PCR for Detecting Class 1, 2 and 3 Integrons. *Journal of Antimicrobial Chemotherapy* **2010**, *65* (8), 1642–1645. https://doi.org/10.1093/jac/dkq167.

(25) Araujo, R. V.; Feitosa-Suntheimer, F.; Gold, A. S.; Londono-Renteria, B.; Colpitts, T. M. One-Step RT-QPCR Assay for ZIKV RNA Detection in Aedes Aegypti Samples: A Protocol to Study Infection and Gene Expression during ZIKV Infection. *Parasit Vectors* **2020**, *13* (1), 128. https://doi.org/10.1186/s13071-020-4002-x.

(26) Tambalo, D. D.; Boa, T.; Liljebjelke, K.; Yost, C. K. Evaluation of Two Quantitative PCR Assays Using Bacteroidales and Mitochondrial DNA Markers for Tracking Dog Fecal Contamination in Waterbodies. *J Microbiol Methods* **2012**, *91* (3), 459–467. https://doi.org/10.1016/j.mimet.2012.09.029.

(27) Knee, J.; Sumner, T.; Adriano, Z.; Anderson, C.; Bush, F.; Capone, D.; Casmo, V.; Holcomb, D.; Kolsky, P.; MacDougall, A.; Molotkova, E.; Braga, J. M.; Russo, C.; Schmidt, W. P.; Stewart, J.; Zambrana, W.; Zuin, V.; Nalá, R.; Cumming, O.; Brown, J. Effects of an Urban Sanitation Intervention on Childhood Enteric Infection and Diarrhea in Maputo, Mozambique: A Controlled before-and-after Trial. *Elife* **2021**, *10*. https://doi.org/10.7554/eLife.62278.
