# Supplementary material for: Environmental pathogen surveillance in cities without universal piped wastewater infrastructure
Source: PLOS Glob Public Health. 2026 Apr 10;6(4):e0004994. doi: 10.1371/journal.pgph.0004994 (PMC13068267; doi:10.1371/journal.pgph.0004994)
Supplement: S2 Table — (PDF) [file pgph.0004994.s007.pdf]

S2 Table. Standard Curve

Target	Intercept	Slope	R^2	Efficiency (%)	Low.95 (gc/rxn)	LOD (gc/rxn)	LOQ (gc/rxn)
**16S pan**	31.6	-1.890	0.8360	116	0.1	NA	0.1
**18s (manufacturer control)**	NA						
**adenovirus 40/41**	34.4	-3.313	0.9968	100.4	0.0	NA	0.001
***Aeromonas* spp.**	35.3	-3.383	0.9987	97.5	10.0	6.98	6.98
Ancylostoma duodenale	36.6	-3.359	0.9973	98.5	3.2	1.52	212
Ascaris lumbricoides	35.8	-3.407	0.9987	96.6	100.0	8.66	240
**astrovirus**	34.2	-3.368	0.9971	98.1	10.0	3.63	152
**BHV**	36.7	-3.409	0.9992	96.5	100.0	5.38	65.0
**BRSV**	34.4	-3.261	0.9956	102.6	3.2	5.13	5.13
Clostridium difficile	34.5	-3.367	0.9983	98.1	10.0	1.78	12.0
Campylobacter jejuni/coli	35.5	-3.408	0.9986	96.5	10.0	6.48	500
***Cryptosporidium* spp.**	36.0	-3.407	0.9987	96.6	10.0	6.50	Inf
**EAEC_(*aaiC*)**	35.9	-3.436	0.9989	95.4	10.0	7.44	7.44
**EAEC_(*aatA*)**	35.0	-3.404	0.9961	96.7	10.0	5.32	2832
**EIEC / *Shigella***	35.4	-3.425	0.9974	95.9	10.0	3.49	311
**EPEC (*bfpA*)**	36.1	-3.490	0.9979	93.4	10.0	9.54	Inf
**EPEC (*eae*)**	34.4	-3.354	0.9966	98.7	3.2	3.04	6.00
**ETEC (*LT*)**	35.8	-3.399	0.9991	96.9	3.2	3.78	301
**ETEC (*STh*)**	35.4	-3.408	0.9985	96.5	3.2	2.40	93
**ETEC (*STp*)**	35.9	-3.187	0.9892	106.0	10.0	NA	1159
**E. coli O157:H7**	35.1	-3.368	0.9979	98.1	3.2	2.70	849
Entamoeba histolytica	36.3	-3.442	0.9975	95.2	100.0	7.17	108
***Giardia* spp.**	35.1	-3.365	0.9986	98.2	10.0	6.44	2250
**HIV**	36.6	-3.410	0.9985	96.4	10.0	4.83	3808
Helicobacter pylori	35.4	-3.468	0.9977	94.3	10.0	4.30	689
**Human mtDNA**	34.9	-3.354	0.9974	98.7	3.2	1.87	165

Necator americanus	36.2	-3.437	0.9986	95.4	3.2	1.94	1596
**norovirus GI**	33.5	-3.325	0.9991	99.9	10.0	3.49	12
**norovirus GII**	36.2	-3.342	0.9974	99.2	3.2	3.33	131
**rotavirus**	35.6	-3.395	0.9967	97.0	10.0	3.76	62
**SARS-COV-2 (N1)**	35.7	-3.373	0.9977	97.9	10.0	4.48	Inf
**STEC (*stx1*)**	35.8	-3.431	0.9989	95.7	3.2	3.78	540
**STEC (*stx2*)**	33.3	-2.950	0.9620	118.3	0.1	NA	0.1
***Salmonella* spp. (*ttr*)**	36.0	-3.428	0.9979	95.8	100.0	9.18	5604
**sapovirus**	37.3	-3.190	0.9953	105.8	10.0	9.59	5336
Trichuris trichiura	34.2	-3.307	0.9980	100.6	1.0	0.50	0.501
Vibrio cholerae (hylA)	32.4	-3.254	0.9876	102.9	10.0	2.79	914
**Avian 16s**	35.8	-3.403	0.9961	96.7	10.0	12.91	1083
**Canine mtDNA**	35.7	-3.246	0.9934	103.3	10.0	6.95	336
**EAEC (*aggR*)**	35.6	-3.411	0.9989	96.4	3.2	3.35	294
**Hepatitis G**	35.6	-3.344	0.9953	99.1	10.0	NA	2.00
**intl1**	33.6	-3.412	0.9978	96.4	10.0	2.06	4.00
***Leptospira* spp.**	37.5	-3.363	0.9977	98.3	3.2	1.62	375
Plasmodium* spp.	36.5	-3.404	0.9968	96.7	3.2	1.99	78
Mycobacterium tuberculosis	36.9	-3.367	0.9970	98.2	10.0	4.95	4.95
**Poultry mtDNA**	36.0	-3.411	0.9975	96.4	10.0	16.18	9314
***Toxocara* spp.**	35.9	-3.420	0.9982	96.1	100.0	12.26	58.0
**zika**	33.4	-3.239	0.9950	103.6	10.0	6.94	6.94

*Excluded from analyses due to apparent cross-reactivity. Low.95 is the lowest standard with at least 95% positive detection. LOD is the 95% limit of detection as determined by probit modeling. LOQ is the limit of quantification as determined by decay modeling, using the user-selected CV threshold of 0.35. TAC reaction wells have a volume of 1.5 µL, comprising 0.6 µL of template and 0.9 µL of mastermix. Gene copies per reaction (gc/rxn).
