# Supplementary material for: Environmental pathogen surveillance in cities without universal piped wastewater infrastructure
Source: PLOS Glob Public Health. 2026 Apr 10;6(4):e0004994. doi: 10.1371/journal.pgph.0004994 (PMC13068267; doi:10.1371/journal.pgph.0004994)
Supplement: S3 Table — (PDF) [file pgph.0004994.s008.pdf]

S3 Table. MIQE Checklist

ITEM TO CHECK	IMPORTANCE	CHECKLIST
**EXPERIMENTAL DESIGN**		
Definition of experimental and control groups	**E**	Cross-sectional study with no intervention or control group
Number within each group	**E**	WWTP Influent: n=10<br>Fecal Sludge: n=24<br>Wastewater surface discharge: n=12<br>WWTP effluent: n=10<br>Open drain: n=34<br>Surface water: n=24<br>Stormwater: n=6
Assay carried out by core lab or investigator's lab?	D	Investigator's lab
**SAMPLE**		
Description	**E**	Approximately 6 liters of water was concentrated to 6mL using the BMFS system. We extracted from 200uL of this pellet for BFMS samples and directly from 200uL of sludge for fecal sludge grab samples.
Volume/mass of sample processed	D	200uL
Microdissection or macrodissection	**E**	Not applicable
Processing procedure	**E**	Initially frozen at -20C on same day as collection. Shipped on dry ice and stored at -80C.
Sample storage conditions and duration (especially for FFPE samples)	**E**	Samples were stored at -20C for approximately six months and at -80C for approximately nine months.
**NUCLEIC ACID EXTRACTION**		
Procedure and/or instrumentation	**E**	See methods section
Name of kit and details of any modifications	**E**	QIAamp 96 Virus QIAcube HT Kit automated on a QIAcube HT
Source of additional reagents used	D	Qiagen Powerbead Pro Tubes
Details of DNase or RNAse treatment	**E**	Not applicable
Contamination assessment (DNA or RNA)	**E**	At least one extraction negative control was included during each day of extractions
Nucleic acid quantification	**E**	Qubit 1X HS dsDNA Kit

Instrument and method	**E**	Qubit 4 Fluorometer
RNA integrity method/instrument	**E**	Not measured
Inhibition testing (Cq dilutions, spike or other)	**E**	Monitored amplification of spiked controls
**REVERSE TRANSCRIPTION**		
Complete reaction conditions	**E**	One-step reverse transcription
Amount of RNA and reaction volume	**E**	Reaction Volume = 1.5 µL
Priming oligonucleotide (if using GSP) and concentration	**E**	Proprietary
Reverse transcriptase and concentration	**E**	ArrayScript™ Reverse Transcriptase
Temperature and time	**E**	45°C for 20 minutes
Manufacturer of reagents and catalogue numbers	D	Applied Biosystems, AgPath-ID™ One-Step RT-PCR Reagents, Catalog number: 4387391
**qPCR TARGET INFORMATION**		
If multiplex, efficiency and LOD of each assay.	**E**	Table S1
Location of amplicon	D	See references in Table S1
In silico specificity screen (BLAST, etc)	**E**	We BLASTed all assays to confirm specificity before ordering the custom TAC.
**qPCR OLIGONUCLEOTIDES**		
Primer sequences	**E**	See references in Table S1
Probe sequences	D**	See references in Table S1
Location and identity of any modifications	**E**	No modifications
Manufacturer of oligonucleotides	D	ThermoFisher Scientific
**qPCR PROTOCOL**		
Complete reaction conditions	**E**	45°C for 20 min and 95°C for 10 min, followed by 45 cycles of 95°C for 15 s and 60°C for 1 min
Reaction volume and amount of cDNA/DNA	**E**	40 µL of template with 60 µL of AgPath-ID™ One-Step RT-PCR Reagents
Primer, (probe), Mg++ and dNTP concentrations	**E**	All assays contained the same concentrations of primers (900 nanomolar) and probe (250 nanomolar). The Mg2+ and dNTP concentrations are not listed in the User Guide.

Polymerase identity and concentration	**E**	AmpliTaq Gold™ polymerase
Buffer/kit identity and manufacturer	**E**	AgPath-ID™ One-Step RT-PCR Reagents
Additives (SYBR Green I, DMSO, etc.)	**E**	No additives
Manufacturer of plates/tubes and catalog number	D	ThermoFisher Scientific
Complete thermocycling parameters	**E**	45°C for 20 min and 95°C for 10 min, followed by 45 cycles of 95°C for 15 s and 60°C for 1 min
Reaction setup (manual/robotic)	D	Manual set-up in a disinfected dead air box (10% bleach with fifteen minutes of contact time, UV for fifteen minutes, and a final cleaning step with 70% ethanol)
Manufacturer of qPCR instrument	**E**	ThermoFisher Scientfic
**qPCR VALIDATION**		
Evidence of optimisation (from gradients)	D	See Liu *et al*. 2013 and Liu *et al*. 2016
Specificity (gel, sequence, melt, or digest)	**E**	See Liu *et al*. 2013 and Liu *et al*. 2016.
Standard curves with slope and y-intercept	**E**	See references in Table S2
PCR efficiency calculated from slope	**E**	See references in Table S2
r2 of standard curve	**E**	See references in Table S2
Evidence for limit of detection	**E**	See references in Table S2
**DATA ANALYSIS**		
qPCR analysis program (source, version)	**E**	QuantStudio Real-Time PCR Software V1.2 CDC
Cq method determination	**E**	Manual thresholding
Results of NTCs	**E**	We observed no amplification before at Ct of 40 in our negative controls. However, we did observe unexpected amplification in the *Plasmodium* assay and this was excluded from the manuscript
Justification of number and choice of reference genes	**E**	
Description of normalisation method	**E**	Volumetric normalization for water samples and per gram dry soil for soil samples.
Software (source, version)	E	R Studio V4.4.0

Note: minimum information for publication of quantitative real-time PCR experiments (MIQE)
