# Supplementary material for: Environmental pathogen surveillance in cities without universal piped wastewater infrastructure
Source: PLOS Glob Public Health. 2026 Apr 10;6(4):e0004994. doi: 10.1371/journal.pgph.0004994 (PMC13068267; doi:10.1371/journal.pgph.0004994)
Supplement: S4 Table — (PDF) [file pgph.0004994.s009.pdf]

S4 Table. Precipitation and temperature

Matrix	Target	Precipitation (mm, z-score increase) Log_{10} (95% confidence interval)	Temperature (°C, z-score increase) Log_{10} (95% confidence interval)
WW Influent	Pooled pathogen	-0.33 (-0.56, -0.11)	0.03 (-0.10, 0.15)
	Pooled bacteria	-0.21 (-0.40, -0.02)	-0.11 (-0.22, 0.0)
	Pooled protozoa	-0.98 (-1.82, -0.19)	0.06 (-0.37, 0.49)
	Pooled virus	-0.26 (-0.88, 0.35)	0.32 (-0.04, 0.68)
	Pooled FST	-0.35 (-0.79, 0.12)	-0.15 (-0.39, 0.11)
Fecal Sludge	Pooled pathogen	0.15 (-0.25, 0.54)	0.28 (0.06, 0.51)
	Pooled bacteria	0.45 (-0.24, 1.1)	0.27 (-0.09, 0.65)
	Pooled protozoa	-0.16 (-0.81, 0.43)	0.16 (-0.19, 0.51)
	Pooled virus	-0.13 (-0.90, 0.62)	0.33 (-0.08, 0.76)
	Pooled FST	0.31 (-0.28, 0.96)	0.08 (-0.24, 0.40)
WW Effluent	Pooled pathogen	-0.08 (-0.23, 0.07)	0.10 (0.0, 0.20)
	Pooled bacteria	0.03 (-0.21, 0.27)	0.23 (0.07, 0.39)
	Pooled protozoa	-0.35 (-0.79, 0.08)	-0.25 (-0.53, 0.04)
	Pooled virus	-0.13 (-0.32, 0.06)	0.08 (-0.04, 0.21)
	Pooled FST	0.09 (-0.42, 0.58)	-0.07 (-0.43, 0.22)
River Water	Pooled pathogen	-0.42 (-0.73, -0.13)	0.23 (0.09, 0.40)
	Pooled bacteria	-0.28 (-0.65, 0.08)	0.29 (0.07, 0.50)
	Pooled protozoa	-0.90 (-1.9, 0.03)	0.18 (-0.59, 0.66)
	Pooled virus	-0.34 (-1.0, 0.29)	0.24 (-0.14, 0.67)
	Pooled FST	0.10 (-0.35, 0.60)	0.24 (-0.07, 0.58)
Open Drains	Pooled pathogen	0.26 (0.16, 0.37)	-0.16 (-0.30, -0.03)
	Pooled bacteria	0.37 (0.24, 0.51)	-0.26 (-0.44, -0.09)
	Pooled protozoa	0.18 (-0.10, 0.49)	-0.22 (-0.59, 0.15)
	Pooled virus	0.27 (-0.02, 0.55)	-0.37 (-0.78, 0.0)
	Pooled FST	0.16 (-0.06, 0.38)	-0.71 (-1.0, -0.45)
Outfalls	Pooled pathogen	-0.18 (-0.52, 0.15)	0.14 (-0.17, 0.43)
	Pooled bacteria	-0.22 (-0.65, 0.21)	0.28 (-0.10, 0.65)
	Pooled protozoa	-0.35 (-0.88, 0.23)	-0.55 (-1.2, 0.01)
	Pooled virus	-0.10 (-0.83, 0.63)	0.19 (-0.46, 0.83)

	Pooled FST	-0.12 (-1.2, 1.0)	-0.19 (-1.2, 0.84)
