# Supplementary material for: Environmental pathogen surveillance in cities without universal piped wastewater infrastructure
Source: PLOS Glob Public Health. 2026 Apr 10;6(4):e0004994. doi: 10.1371/journal.pgph.0004994 (PMC13068267; doi:10.1371/journal.pgph.0004994)
Supplement: S5 Table — (PDF) [file pgph.0004994.s010.pdf]

S5 Table. Pediatric infection comparison

	WWTP Influent	Fecal Sludge	WW Outfall	WWTP Effluent	Open Drain	River Water	Flood Water	Child Feces
Target	This study							MapSan Trial 24-month follow up[27]
16S rRNA	100% (10/10)	100% (24/24)	100% (12/12)	100% (10/10)	100% (34/34)	100% (24/24)	100% (6/6)	NA
A. duodenale	0% (0/10)	8.3% (2/24)	0% (0/12)	0% (0/10)	0% (0/34)	0% (0/24)	0% (0/6)	NA
A. lumbricoides	0% (0/10)	0 (0/24)	0% (0/12)	0% (0/10)	0% (0/34)	0% (0/24)	0% (0/6)	25% (134/545)
Adenovirus 40/41	100% (10/10)	25% (6/24)	83% (10/12)	100% (10/10)	62% (21/34)	46% (11/24)	0% (0/6)	1% (13/921)
Aeromonas	100% (10/10)	71% (17/24)	100% (12/12)	100% (10/10)	100% (34/34)	100% (24/24)	83% (5/6)	NA
Astrovirus	100% (10/10)	54% (13/24)	92% (11/12)	100% (10/10)	41% (14/34)	25% (6/24)	0% (0/6)	NA
Avian 16S	0% (0/10)	0% (0/24)	0% (0/12)	0% (0/10)	5.9% (2/34)	0% (0/24)	50% (3/6)	NA
C. difficile	70% (7/10)	0% (0/24)	8.3% (1/12)	10% (1/10)	5.9% (2/34)	0% (0/24)	0% (0/6)	3% (24/921)
C. jejuni/coli	90% (9/10)	4.2% (1/24)	67% (8/12)	20% (2/10)	18% (6/34)	4.2% (1/24)	0% (0/6)	9% (82/921)
Canine mtDNA	20% (2/10)	8.3% (2/24)	0% (0/12)	10% (1/10)	18% (6/34)	4/2% (1/24)	0.17% (1/6)	NA
Cryptosporidium	70% (7/10)	50% (12/24)	50% (6/12)	100% (10/10)	56% (19/34)	83% (20/24)	83% (5/6)	3% (29/921)
E. coli O157:H7	100% (10/10)	4.2% (1/24)	8.3% (1/12)	10% (1/10)	15% (5/34)	4.2% (1/24)	0% (0/6)	4% (41/921)
E. histolytica	90% (9/10)	8.3 (2/24)	42% (5/12)	80% (8/10)	12% (4/34)	8.3% (2/24)	0% (0/6)	NA
EAEC	100% (10/10)	38% (9/24)	100% (12/12)	100% (10/10)	79% (27/34)	17% (4/24)	17% (1/6)	NA
EPEC	100% (10/10)	46% (11/24)	100% (12/12)	100% (10/10)	62% (21/34)	21% (5/24)	33% (2/6)	NA
ETEC	100% (10/10)	33% (8/24)	50% (6/12)	100% (10/10)	44% (15/34)	17% (4/24)	0% (0/6)	27% (253/921)
Giardia	100% (10/10)	83% (20/24)	92% (11/12)	100% (10/10)	32% (11/34)	8.3% (2/24)	17% (1/6)	63% (583/921)
H. pylori	0% (0/10)	0% (0/24)	0% (0/12)	0% (0/10)	0% (0/34)	0% (0/24)	0% (0/6)	NA
HIV	0% (0/10)	0% (0/24)	0% (0/12)	0% (0/10)	0% (0/34)	0% (0/24)	0% (0/6)	NA
human mtDNA	100% (10/10)	100% (24/24)	100% (12/12)	100% (10/10)	85% (29/34)	54% (13/24)	83% (5/6)	NA
intl1	100% (10/10)	100% (24/24)	100% (12/12)	100% (10/10)	100% (34/34)	100% (24/24)	100% (6/6)	NA
Leptospira	0% (0/10)	4.2% (1/24)	0% (0/12)	0% (0/10)	0% (0/34)	0% (0/24)	0% (0/6)	NA
M. tuberculosis	0% (0/10)	0% (0/24)	0% (0/12)	10% (1/10)	0% (0/34)	0% (0/24)	0% (0/6)	NA
N. americanus	0% (0/10)	4.2% (1/24)	0% (0/12)	0% (0/10)	0% (0/34)	0% (0/24)	0% (0/6)	NA
Norovirus	100% (10/10)	75% (18/24)	100% (12/12)	100% (10/10)	53% (18/34)	38% (9/24)	0% (0/6)	11% (102/921)
Poultry mtDNA	60% (6/10)	29% (7/24)	25% (3/12)	30% (3/10)	32% (11/34)	4.2% (1/24)	17% (1/6)	NA
Rotavirus	90% (9/10)	38% (9/24)	83% (10/12)	100% (10/10)	50% (17/34)	33% (8/24)	17% (1/6)	NA
Salmonella	60% (6/10)	0% (0/24)	33% (4/12)	0.2 (2/10)	0% (0/34)	0% (0/24)	0% (0/6)	NA
Sapovirus	90% (9/10)	54% (13/24)	83% (10/12)	100% (10/10)	32% (11/34)	8.3% (2/24)	17% (1/6)	NA

SARS-CoV-2	40% (4/10)	13% (3/24)	0% (0/12)	0.1 (1/10)	2.9% (1/34)	0% (0/24)	0% (0/6)	NA
Shigella / EIEC	100% (10/10)	17% (4/24)	92% (11/12)	90% (9/10)	44% (15/34)	4.2% (1/24)	0% (0/6)	56% (514/921)
STEC	80% (8/10)	4% (1/24)	25% (3/12)	30% (3/10)	15% (5/34)	0% (0/24)	0% (0/6)	35 (32/921)
T. trichiura	10% (1/10)	0% (0/24)	0% (0/12)	0% (0/10)	18% (6/34)	13% (3/24)	0% (0/6)	44% (241/545)
Toxocara spp.	0% (0/10)	0 (0/24)	0% (0/12)	0% (0/10)	0% (0/34)	0 (0/24)	0% (0/6)	NA
Vibrio spp.	100% (10/10)	13% (3/24)	83% (10/12)	70% (7/10)	82% (28/34)	54% (13/24)	83% (5/6)	NA
Zika	10% (1/10)	33% (8/24)	0% (0/12)	0% (0/10)	8.8% (3/34)	0% (0/24)	0% (0/6)	NA
