# Supplementary material for: Environmental pathogen surveillance in cities without universal piped wastewater infrastructure
Source: PLOS Glob Public Health. 2026 Apr 10;6(4):e0004994. doi: 10.1371/journal.pgph.0004994 (PMC13068267; doi:10.1371/journal.pgph.0004994)
Supplement: S6 Table — (PDF) [file pgph.0004994.s011.pdf]

S6 Table. Use cases

**Use Case**	**Description**	**Target category**	**Potential Targets**	**Methods**
To inform local and global eradication efforts	The presence of these targets could trigger a mass vaccination campaign in the project area	Endemic pathogens	poliovirus, measles virus	PCR
To inform routine vaccination	These targets can be evaluated longitudinally to assess the impact of routine vaccination campaigns on their presence and trends in our samples by identifying locations with prevalent infections in the absence of clinical data, and facilitating an understanding on the impact of vaccination on virus circulation	Endemic pathogens	hepatitis A virus, poliovirus, rotavirus, *Salmonella* Typhi, and other vaccine candidates	PCR
		Epidemic and emerging pathogens	SARS-CoV-2 and its variants; Zika virus; other emerging, re-emerging, and future novel pathogens with outbreak potential	
Baseline data to evaluate new vaccines	As new vaccines become available longitudinal data can enable analysis of their impact at a community level	Endemic pathogens	*Shigella* spp., *Necator americanus*, other vaccine candidates	PCR
To evaluate Kenya's helminth mass drug administration (MDA) program	School age children are dewormed annually in Kenya. Longitudinal data provide the opportunity to evaluate the effectiveness of the MDA program at a community level.	Endemic intestinal worms	*Ascaris lumbricoides*, *Trichuris trichiura*, *Ancylostoma duodenale*, *Necator americanus*	PCR
To inform clinical treatment options	Data regarding what pathogens are most frequently circulating in the community can inform clinical decision making.	Endemic pathogens	Enteric and respiratory pathogens	PCR
	Choice of antibiotics by health care workers can be informed by the presence of AMR at the community level. In addition, use of antibiotics can be informed by the prevalence of circulating bacterial pathogens compared to viral or protozoan pathogens.	Antimicrobial resistance	All antibacterial classes* evaluated by current WHO AMR surveillance programs (e.g., GLASS and Tricycle Project).	Culture<br>Metagenomics<br>PCR
Pathogen, Variant, and AMR control:<br>Is the target present in the community?	The presence of a specific target of concern can raise community awareness, especially as new variants emerge, or resistant strains increase that may require changes to public health response.	Epidemic and emerging pathogens	SARS-CoV-2 and its variants; Zika virus; other emerging, re-emerging, and future novel pathogens with outbreak potential	Metagenomics<br>PCR
		Endemic pathogens	Enteric and respiratory pathogens	PCR

		Antimicrobial resistance	All antibacterial classes* evaluated by current WHO AMR surveillance programs (e.g., GLASS and Tricycle Project)	Culture<br>Metagenomics<br>PCR
Pathogen, Variant, and AMR control: Tracking increasing or decreasing trends at a community level	Increasing or decreasing trends of pathogen, variant or AMR targets at a community level provides an early warning of spread or reduction of disease within the community. This advanced notice enables deployment of interventions to reduce transmission, clinical preparedness for increasing cases, and advises health care workers which pathogens are most likely to present in a clinical setting.	Epidemic and emerging pathogens	SARS-CoV-2 and its variants; Zika virus; other emerging, re-emerging, and future novel pathogens with outbreak potential	Metagenomics<br>PCR
		Endemic pathogens	Enteric and respiratory pathogens	PCR
		Antimicrobial resistance	All antibacterial classes* evaluated by current WHO AMR surveillance programs (e.g., GLASS and Tricycle Project)	Culture<br>Metagenomics<br>PCR
Evaluation of public health interventions	Longitudinal data across multiple geographic areas will enable assessment of interventions compared to control areas that did not receive the interventions. These interventions could include infrastructure improvements, behavior change interventions, or policy changes.	Epidemic and emerging pathogens	SARS-CoV-2 and its variants; Zika virus; other emerging, re-emerging, and future novel pathogens with outbreak potential	Metagenomics<br>PCR
		Endemic pathogens	Enteric and respiratory pathogens	PCR
		Antimicrobial resistance	All antibacterial classes* evaluated by current WHO AMR surveillance programs (e.g., GLASS and Tricycle Project)	Culture<br>Metagenomics<br>PCR
Promoting healthy behaviors	Data dashboards can inform health care workers and potentially community members on which pathogens are circulating in the community. These data may encourage people to follow public health guidance and reduce their risk of infection. Examples of public health guidance include clinical or personal testing, compliance with control interventions, and vaccine uptake.	Epidemic and emerging pathogens	SARS-CoV-2 and its variants; Zika virus; other emerging, re-emerging, and future novel pathogens with outbreak potential	Metagenomics<br>PCR
		Endemic pathogens	Enteric and respiratory pathogens	PCR
		Antimicrobial resistance	All antibacterial classes* evaluated by current WHO AMR surveillance programs (e.g., GLASS and Tricycle Project)	Culture<br>Metagenomics<br>PCR

Screening for target shedding in a specific geographic area, most notably in communities lacking clinical surveillance.	Identification of geographic hotspots allows health care workers to focus the public health response in priority locations	Epidemic and emerging pathogens	SARS-CoV-2 and its variants; Zika virus; other emerging, re-emerging, and future novel pathogens with outbreak potential	Metagenomics<br>PCR
		Endemic pathogens	Enteric and respiratory pathogens	PCR
		Antimicrobial resistance	All antibacterial classes* evaluated by current WHO AMR surveillance programs (e.g., GLASS and Tricycle Project)	Culture<br>Metagenomics<br>PCR
