# Supplementary material for: Environmental pathogen surveillance in cities without universal piped wastewater infrastructure
Source: PLOS Glob Public Health. 2026 Apr 10;6(4):e0004994. doi: 10.1371/journal.pgph.0004994 (PMC13068267; doi:10.1371/journal.pgph.0004994)
Supplement: S2 Text — (PDF) [file pgph.0004994.s013.pdf]

S2 Text. Detailed methods

Water Samples

We collected and performed primary concentration of water samples (i.e., wastewater, river water, open drain water) using the bag-mediated filtration system (BMFS, Scientific Methods, Granger, Indiana) (S2 Fig).[4–6] The most recent BMFS protocols were shared with the study team by the Meschke Lab at University of Washington.[7] Open bags were dragged through the water source to collect approximately six liters of water. After mounting the bag on a tripod, water was gravity filtered through a ViroCap filter (Scientific Methods, Granger, Indiana).[8] If the entire volume passed through the filter in less than 20 minutes, the process was repeated. If less than six liters passed through the filter in 30 minutes, then this volume was recorded. A 200 mL grab sample was also collected using a Whirl-Pak bag (Nasco, Pleasant Prairie, Wisconsin). We recorded water temperature using a handheld meter and pH using pH strips (VWR, Radnor, PA).

We expected that the solids content of the fecal sludge being deposited at Maputo's WWTP would have rendered BMFS unfeasible due to rapid filter clogging. Instead, we collected a 50 mL grab sample of sludge from the point of discharge (Figure S3) using the Sludge Nabber sampling device (Nasco, Pleasant Prairie, Wisconsin) into a sterile 50 mL centrifuge tube (VWR, Radnor, PA).[9] Sludge samples were not collected directly from trucks discharging fecal waste into the anaerobic pond. Instead, samples were collected directly from the anaerobic pond (see Figure S3).

We used the standard secondary concentration procedure for BMFS from Zhou *et al*. 2018.[6] First, we eluted ViroCap filters with 300 mL of 1.5% (weight/volume) beef extract and 0.05 M glycine solution at pH 9.5 (Becton Dickinson, Franklin Lakes, NJ). The recovered eluate was pH adjusted to 7.0-7.5, 5% skimmed milk was added to achieve a 0.05% final skimmed milk concentration, the mixture was shaken at 200 rpm for 2 hours to form flocs, after which we

centrifuged the mixture at 6000 x *g* at 4°C for 30 minutes. Finally, we resuspended the pellet in 6 ml of autoclaved distilled water.

Culture Methods

We cultured *E. coli* using IDEXX Quanti-tray/2000 system (IDEXX, Westbrook, Maine). Collected water grab samples and 1g of soil samples (wet weight) were diluted in ten-fold serial dilutions, ranging from 1:10 to 1:10^6, combined with Colilert-18 reagent, incubated at 37°C for 18 hours, and fluorescent wells visually counted in a UV reading chamber (IDEXX, Westbrook, Maine). Water samples were diluted such that lower limit of detection (LOD) was 10 most probable number (MPN) *E. coli* per 100 mL water and the upper limit of quantification (ULQ) was 10^8 MPN *E. coli* per 100 mL. Approximately 5g of soil was dried using the microwave oven method to determine the moisture content.[10] The LOD for soil was approximately 100 MPN *E. coli* per gram dry soil and ULQ was 10^8 MPN *E. coli* per gram dry soil. We included one negative control (i.e., distilled water) each day.

Molecular Analysis

Sample aliquots were shipped from Centro de Investigação e Treino em Saúde da Polana Caniço (CISPOC) in Maputo, Mozambique to the University of North Carolina at Chapel Hill on dry ice with temperature monitoring. We extracted nucleic acids from either 200 µL of resuspended pellet or 200 µL of fecal sludge using the Qiagen QIAamp 96 Virus QIAcube HT Kit, which was automated on the QiaCube (Qiagen, Hilden, Germany). Molecular analysis of soil samples is reported separately (Dalton *et al.*, in prep). Pre-treatment was performed using methods previously validated for multi-pathogen PCR, which included bead beating in Qiagen Powerbead Pro tubes for 2 x 5 minutes at 25 Hz using a Qiagen TissueLyser. Live vaccine-derived bovine herpes virus (BHV, a DNA virus) and bovine respiratory syncytial virus (BRSV,

an RNA virus) were used as extraction positive controls, while molecular water was used for extraction negative controls[11]. The BHV and BRSV were spiked directly in bead beating tubes with the sample during extraction. We included at least one negative extraction control each day of extractions. Following extraction, template from each sample underwent inhibitor removal using the Zymo 1-step PCR Inhibitor Removal Kit (Zymo, Irvine, California).

We analyzed purified nucleic acids for enteric pathogen genes and microbial source tracking markers (MST) using reverse transcription quantitative PCR (RT-qPCR) via a custom TaqMan Array Card (TAC) (Table S1, Table S2). We selected the targets on the TAC based on our previous work in Maputo and knowledge of which animals are present in the study area. The targets on the TAC included adenovirus 40/41; astrovirus; norovirus GI/GII; rotavirus; sapovirus; SARS-CoV-2; *Aeromonas* spp.; *Campylobacter jejuni*/*coli*; *E. coli* O157; *Clostridium difficile*, toxin B; enteroaggregative *E. coli* (EAEC); shiga-like toxin producing *E. coli* (STEC); enteropathogenic *E. coli* (EPEC); enterotoxigenic *E. coli* (ETEC); *Helicobacter pylori*; *Plesiomonas shigelloides*; *Shigella* spp.; *Salmonella enterica*; *Vibrio cholerae*; *Yersinia enterocolitica*; *Cryptosporidium* spp.; *Entamoeba histolytica; Giardia* spp.; *Ascaris lumbricoides*; *Trichuris trichiura*; *Ancylostoma duodenale*; *Necator americanus*, *Strongyloides stercolaris*.[12] MST markers included human mtDNA[13], canine mtDNA[14], poultry mtDNA[15], *Toxocara* spp.[16], and avian 16S rRNA[15]. The class 1 integron-integrase gene was included as a proxy for potential antimicrobial resistance.[17]

<u>Standard curve</u>

We developed an engineered combined positive control for all the targets on our custom TAC following the approach outlined by others[1]. The primers and probe sequences for all targets were concatenated and synthesized on three plasmids (Twist Biosciences, San Francisco, USA),

two for the DNA gene targets and the other the RNA gene targets. Plasmids were linearized with a BshT1 restriction enzyme (Thermo Fisher Scientific, Waltham, MA) and the RNA-target plasmid transcribed with MEGAscript T7 Transcription Kit followed by MEGAclear Transcription Clean-Up Kit (Thermo Fisher Scientific, Waltham, MA) to generate RNA control material. The DNA plasmid concentration (copies/µL) was determined from the manufacturer-provided mass and the transcribed RNA plasmid was quantified using a Qubit RNA HS Assay Kit on Qubit 4 Fluorometer (Thermo Fisher Scientific, Waltham, MA). The plasmids were mixed 1:1 by concentration, aliquoted in 0.6 mL DNAse/RNAase-free microcentrifuge tubes and stored at -80°C for single use to minimize freeze-thaw cycles. We used the combined plasmids at stock concentration on one TAC card per day of analysis as a positive control and as standard reference material to generate standard curves for target quantification. We fit standard curves to nine-point, ten-fold dilution series (10^{8}-10^{-1} gene copies per µL of template) and two five-fold dilution series ($10^{-0.5}$ and $10^{0.5}$) of the engineered combined positive control run on 16 separate TACs to produce the master standard curve. Positive control material concentrations were determined using dPCR on a QIACuity 4 system (Qiagen, Hilden, Germany).

Limit of Detection and Limit of Quantitation

We used empirical experiments of repeated measures in the determination of LODs and LOQs. We ran the following concentrations (gc/µL): 10^{-1}, $10^{-0.5}$, 10^{0}, $10^{0.5}$, 10^{1}, 10^{2} and repeated each concentration for all eight TAC ports. A total of seven TACs were run, ensuring each concentration was run once on each QuantStudio7 machine (n=2). The final data analysis was conducted using the Klymus et al. eDNA calculator.[18]

Calculation of Gene Copies

Cq values from RT-qPCR were transformed into normalized gene copy estimates using standard curves and accounting for the dilutions during sample processing and analysis.

Multiple Gene Targets

We included multiple assays for several of the enteric pathogens that we assessed. For example, we assayed the *stx1* and *stx2* genes corresponding to Shiga toxin producing *E. coli*. For each of these pathogens, if any single gene target was positive, then we called that sample positive for that target. If multiple targets were positive, then we only used the largest value in our summary statistics and regression models.

Controls

Dilutions for culture-based analysis using IDEXX were made with autoclaved distilled water. Each day after samples processing 100 mL of sterile autoclaved distilled water was analyzed using IDEXX as a negative control. No contamination was observed in these negative controls.

We created five sample treatment negative controls by filtering 3 liters of autoclaved distilled water in the lab in Mozambique. The sample treatment negative controls were negative for all targets. However, all five were positive for 16S rRNA at low concentrations. We also ran five negative extraction controls and eight negative PCR controls. These thirteen controls were negative for all targets. However, all thirteen were positive for 16S rRNA, which amplified within one Cq of the y-intercept of the standard curve. We were also unable to calculate a limit of detection due for 16S rRNA due to detection at concentrations <1 gc per reaction in our dilution series (see Table S2). *E. coli* is used to manufacture the mastermix we used in this study and low concentrations of *E. coli* genes have been detected in master mix, which may explain the persistent detection of 16S rRNA in our negative controls.

The results of our extraction controls indicate that little to no inhibition was present in our molecular analysis. In our five negative extraction controls the mean Cq value for BRSV was 23.8 (sd=0.31). Among all samples the mean Cq for BRSV was 24.1 (sd=1.1). For BHV was the mean Cq in the negative extraction controls was 29.1 (sd=0.75) while the mean was 26.5 (sd=1.7) in our samples.

<u>Multi-target Analysis</u>

We conducted analyses using Bayesian censored regression (*brms* package), which was calculated as a weighted average over all the pathogen targets within a given class (i.e., separately for all pathogens, bacterial pathogens, viral pathogens, protozoan pathogens, and fecal source tracking markers), as well as for individual pathogens and markers. Models were estimated in brms using four Markov chains, 2000 iterations per chain (1000 warmup) and cores = 4. Default brms priors were used (flat population-level priors; Student-t priors for intercept and scale parameters; LKJ(1) for correlations). The following targets were included in the pooled analyses.

Pooled category	Targets
Pooled pathogen	*A. duodenale, A. lumbricoides*, adenovirus 40/41, *Aeromonas*, astrovirus, *C. difficile*, *C. jejuni/coli*, *Cryptosporidium*, *E. coli* O157:H7, *E. histolytica*, EAEC, EPEC, ETEC, *Giardia*, *H. pylori*, HIV, *Leptospira*, *M. tuberculosis*, *N. americanus*, norovirus, rotavirus, *Salmonella*, sapovirus, SARS-CoV-2, *Shigella*, STEC, *T. trichiura, Toxocara, Vibrio*, Zika
Pooled protozoan pathogen	*Cryptosporidium*, *E. histolytica*, *Giardia*,
Pooled bacterial pathogen	*Aeromonas*, *C. difficile*, *C. jejuni/coli*, *E. coli* O157:H7, EAEC, EPEC, ETEC, *H. pylori*, *Leptospira*, *M. tuberculosis*, *Salmonella*, *Shigella*, STEC*, Vibrio*
Pooled viral pathogen	adenovirus 40/41, astrovirus, HIV, norovirus, rotavirus, sapovirus, SARS-CoV-2, Zika

Pooled fecal source tracking marker	human mtDNA, poultry mtDNA, canine mtDNA, Avian 16S rRNA

R Code

The following R code was used to run the multi-pathogen model:

```
model_pathogen <- brm(conc_LOD_log10 | cens(censored_brm) ~ Category + precip7_mmz +
temp_cz + (1 + Category + 1|targetname),
        data = tacdata_path,
        family = gaussian(), chains=4, cores=4)
summary(model_pathogen)
```
