# Supplementary material for: Environmental pathogen surveillance in cities without universal piped wastewater infrastructure
Source: PLOS Glob Public Health. 2026 Apr 10;6(4):e0004994. doi: 10.1371/journal.pgph.0004994 (PMC13068267; doi:10.1371/journal.pgph.0004994)
Supplement: S3 Text — (PDF) [file pgph.0004994.s014.pdf]

S3 Text. Pathogen Flow Table

We developed pathogen flow tables as a visual representation of enteric pathogens in the domestic domain. Log_{10}-tranformed gene copy concentrations are visualized for commonly detected enteric pathogens in Figure 3 for each environmental matrix assessed. The y-axis is log_{10} transformed gene copy concentrations per liter. Points represent individual samples. The color of each point is a gradation from green to red gradient corresponding to the percent of fecal waste in that matrix that is safely managed according to our estimate based on Maputo's SFD (see table below). Points below the dotted line (i.e. the LOD) are imputed for visualization purposes; the LODs for water samples and for fecal sludge are different and have different visualized LODs in the figure. Data was imputed for non-detects by randomly selecting a log_{10} transformed value from zero to the LOD.

Matrix	% Safely Managed According to the SFD	Description
WWTP Influent	75%	Maputo's SFD visualizes that wastewater from System 1 flows into the WWTP (Not Treated and Adequately Treated Boxes), with 75% (3% of total excreta) receiving adequate treatment and 25% (1% of total excreta) discharging to receiving waters.
Fecal Sludge	52%	88% of excreta is contained in onsite sanitation systems in Maputo. The SFD visualizes that 43% of this is safely managed, estimating that 52% overall is safely managed.
WW Surface Discharge	0%	Maputo's SFD visualizes that all the wastewater from System 2 flows into the drainage system (i.e., outfalls in Maputo Bay).
WWTP Effluent	0%	As visualized on the SFD, WWTP effluent discharges directly into receiving water and
Open Drain	0%	Open drains, visualized by the drainage system bubble on the SFD, are considered 0% safely managed.

River Water	0%	River water, visualized by the receiving waters bubble on the SFD, are considered 0% safely managed.
Stormwater	0%	Stormwater is not visualized on the SFD.

Table. Description of calculation of percent safely managed from Maputo's SFD
