# Supplementary material for: Environmental pathogen surveillance in cities without universal piped wastewater infrastructure
Source: PLOS Glob Public Health. 2026 Apr 10;6(4):e0004994. doi: 10.1371/journal.pgph.0004994 (PMC13068267; doi:10.1371/journal.pgph.0004994)
Supplement: S4 Text — (PDF) [file pgph.0004994.s015.pdf]

S4 Text. Sample Size Calculation

We ran simulations in R to estimate the sample size necessary to determine significant reductions in target concentrations (80% power, α=0.05) for a hypothetical before-and-after analysis for five commonly detected targets.

	Sample Size for $\geq$0.50 $\log_{10}$ reduction in*				
Matrix	MPN *E. coli*	human mtDNA	EAEC	*Giardia*	norovirus
WWTP Influent	15	54	14	87	51
Fecal Sludge (from WWTP)	33	54	NA	403	780
WWTP Effluent	17	19	13	59	6
Open Drains	120	286	359	NA	246
River Water (Site downstream from WWTP)	17	47	601	NA	54
River Water (all samples)	186	326	NA	NA	NA
Flood Water	286	59	NA	NA	NA
Outfalls	30	35	65	107	58

Table S5. Sample size to achieve $\geq$0.50 $\log_{10}$ reduction in target based on the mean and standard deviation observed in our samples with 80% power and α=0.05 for a before-and-after analysis. NA indicates mean concentration was <LOD and sample size calculation was not attempted. Abbreviations: Enteroaggregative *E. coli* (EAEC)

R Code Used

```
#Sample size simulator for manuscript
from_n <- 1
to_n<- 100
by_n <- 1
possible.ns <- seq(from=from_n, to=to_n, by=by_n)     # The sample sizes we'll be considering
powers <- rep(NA, length(possible.ns))           # Empty object to collect simulation estimates
alpha <- 0.05                                    # Standard significance level
sims <- 5000                                     # Number of simulations to conduct for each N
mean_log10 <- 4.5
sd_log10 <- 0.64
#### Outer loop to vary the number of subjects ####
for (j in 1:length(possible.ns)){
  N <- possible.ns[j]                            # Pick the jth value for N
```

```
  significant.experiments <- rep(NA, sims)         # Empty object to count significant experiments

  #### Inner loop to conduct experiments "sims" times over for each N ####
  for (i in 1:sims){
    Y0 <-  rnorm(n=N, mean=mean_log10, sd=sd_log10)  # control potential outcome
    tau <- 0.50                                # Hypothesize treatment effect
    Y1 <- Y0 + tau                             # treatment potential outcome
    Z.sim <- rep(c(0,1), length.out=N)            # Do an assignment evenly split between
intervention and control
    Y.sim <- Y1*Z.sim + Y0*(1-Z.sim)              # Reveal outcomes according to assignment
    fit.sim <- lm(Y.sim ~ Z.sim)                  # Do analysis (Simple regression)
    p.value <- summary(fit.sim)$coefficients[2,4]  # Extract p-values
    significant.experiments[i] <- (p.value <= alpha) # Determine significance according to p <=
0.05
  }

  powers[j] <- mean(significant.experiments)       # store average success rate (power) for each N
}
plot(possible.ns, powers, ylim=c(0,1))
powers
# Find the first index where power > 0.80
index <- which(powers > 0.80)[1]
#Consider starting point to determine first sample with size >80% power
(from_n-(1*by_n)) + index*(by_n)
```
