# Supplementary material for: Environmental pathogen surveillance in cities without universal piped wastewater infrastructure
Source: PLOS Glob Public Health. 2026 Apr 10;6(4):e0004994. doi: 10.1371/journal.pgph.0004994 (PMC13068267; doi:10.1371/journal.pgph.0004994)
Supplement: Attachment — Submitted filename: Response to Reviewers.docx [file pgph.0004994.s017.docx]

Response to Reviewers

| Reviewer 1 |  |  |
| --- | --- | --- |
| Comment | Action Taken | Response |
| Reviewer #1: I have reviewed the manuscript titled "Environmental pathogen surveillance in cities without universal conventional wastewater infrastructure". The manuscript explores the utility of using the wastewater effluents to detect pathogens and form public health response. The work is done in the Maputo region of Mozambique, which is a representative of sewage system present in many cities across the developing world. The work is highly relevant and the manuscript is written in a form that is easy to follow. I have no comments and the manuscript is recommended for acceptance | None needed. | Thank you for your time. |

| Reviewer 2 |  |  |
| --- | --- | --- |
| Comment | Action Taken | Response |
| Reviewer #2: The article is well written, and the authors have described a unique approach to analyze suitability of wastewater matrices by pooling quantitative pathogen detection from different matrices and comparing to the standard of WWTP influents commonly used to predict disease prevalence or outbreaks in high-income countries.  However, there are a few critical gaps in the manuscript, that in its current form fails to explain the intent of the authors especially with regards to the study design, and thorough interpretation of results. | We have revised throughout. | Thank you for your time and detailed review of the manuscript. |
| 1. The authors state in the introduction 3 objectives of which the second was “ Our other goals were to assess presence, concentration, and variance of enteric pathogens in sample types to inform sample size calculations for potential future use cases, to estimate the fraction of pathogens associated with uncontained excreta”. They have not sufficiently described why they wanted to carry this out, and if the study design that they had - “purposive sampling” was sufficient to fulfil this objective. | We added a new paragraph to the introduction.  Line 84:  Empirical data describing the presence, concentration, and variance of enteric pathogens across matrices provides the foundation for developing environmental surveillance systems in cities lacking conventional wastewater infrastructure. Understanding variance and detection frequency for specific pathogens informs sample size calculations and would allow future surveillance efforts to meet minimum probability thresholds for detecting pathogen signals with statistical confidence. Additionally, estimating the proportion of pathogens released into the environment through uncontained excreta helps quantify environmental hazards, support exposure assessment, and prioritize public health interventions most likely to reduce transmission risks. | We agree that additional clarity was necessary for this point in the introduction. |
| 2. The study design should be described in context with the stated objectives - why did they decide on purposive sampling and a justification of why they believe this would be sufficient to address their objectives. | We included two new sections of additional text.  Line 130  We used a purposive approach, as this enabled us to select the most appropriate sampling locations to achieve our objectives.  Line 145  This approach allowed us to include all matrix types, as well as capture the spatial and temporal heterogeneity present in these matrices. | Thank you for this point. We have added additional clarity. |
| 3. The analysis method is fairly well described, however a few more details need to be explained - how where the pathogens weighted in the pooled analysis. Viruses are more easily detected by molecular analysis than bacteria - were the weights based on abundance with more abundant pathogens given less weightage, or potential disease severity? | We added additional description.  Line 228  The weighting was statistical, emerged from the model structure, and was not based on pathogen type or potential disease severity. | We appreciate you brining this to our attention to better clarify. |
| 4. The discussion in the manuscript fails to interpret the results thoroughly and reads like another introduction with recommendations that are not fully supported based on findings from this work. The results from the study based on the mean log difference are not discussed adequately. There is very little discussion on the content and suitability of each matrix for specific pathogens. | We have added a new paragraph to the discussion section.  Line 456  Our results demonstrate that environmental surveillance efforts should prioritize matrices with the highest and most stable concentrations of fecal contamination that best represent the community of interest. Across pathogen classes, pooled and individual estimates were most similar between wastewater influent and fecal sludge, suggesting that fecal sludge – particularly composite samples from holding tanks or sludge discharged at treatment plants – may offer the greatest statistical power for surveillance. However, the utility of fecal sludge as a surveillance matrix depends strongly on the structure of the local fecal sludge disposal chain. In many high-density informal settlements, formal pit emptying is infrequent and sludge is instead buried onsite or removed manually by informal emptiers.^67^ In such contexts, the fecal sludge arriving at a treatment facility may disproportionately represent higher-income neighborhoods, reducing its representativeness for the broader population and limiting its applicability for surveillance. Pathogens were also frequently detected in open drains and river water, but the substantially higher variance in these matrices indicates that a larger number of samples would be required to achieve statistical confidence comparable to that of wastewater influent or fecal sludge. Finally, other matrices not evaluated here (e.g., coprophagous flies^68^) may warrant consideration in low-income communities where aquatic matrices are absent, inaccessible, or unsafe to sample. | We agree that additional discussion was necessary on this point. |
| 5. The results from objective 3 - “fraction of pathogens in uncontained excreta can also be better discussed”. For example, do the results mirror known disease prevalences from Maputo? | Line 479  We compare the pathogens we detect with our previous work in Maputo in Table S5 | We agree this is a very interesting comparison. There are few representative data sources on disease prevalence in Mozambique. We have compared our results with our previous results in Table S5. We agree this is an exciting area of future research and would a compelling use case for this data. |
| 5. The most interesting finding was the increase in detection of bacteria and parasites with increased precipitation- other literature elsewhere (including surveillance in drainage systems) would indicate the opposite due to dilution effects. The authors have addressed this in the discussion and have pointed to possible anthropogenic run-offs that lead to increased detection. Why was a similar increase not seen for viruses or parasites detected? Some more elaboration on this would add much more value to the manuscript. | We completely revised this paragraph in the discussion section.  Line 494:  In urban environments, drainage infrastructure primarily receives runoff from densely populated anthropogenic surfaces such as residential, commercial, and industrial areas, whereas rivers and streams receive runoff from a much broader range of land uses. These differences in hydrology and catchment characteristics may explain why precipitation increased pathogen concentrations in open drains but diluted concentrations in river water. When examined by pathogen class, we observed the same positive association between precipitation and pathogen concentrations for the pooled bacteria estimate but not for protozoa or viruses. This discrepancy may reflect differences in fate and transport among pathogen types, including variation in attachment to particulates, settling behavior, and environmental persistence.^23^ Notably, the dilution of fecal contamination in river water following rainfall contrasts with findings from many high-income settings, where rainfall is typically associated with increased fecal contamination of surface waters. | We agree this was a helpful addition to the manuscript. |
