# Supplementary material for: Environmental pathogen surveillance in cities without universal piped wastewater infrastructure
Source: PLOS Glob Public Health. 2026 Apr 10;6(4):e0004994. doi: 10.1371/journal.pgph.0004994 (PMC13068267; doi:10.1371/journal.pgph.0004994)
Supplement: Attachment — Submitted filename: Response to Reviewers_R2.docx [file pgph.0004994.s018.docx]

Reviewer 3

| Comment | Action Taken | Response |
| --- | --- | --- |
| 1. "conventional" in the title sounds like piped sewer systems with WWTP are the norm globally. However, I do think that unfortunately that is only the case in the global north. Another choice of wording could better reflect this aspect. | We replaced the word conventional with the word piped | Agreed |
| 2. This is a study conducted in Mozambique, with samples collected in Mozambique, and parts of the laboratory analysis performed in the US. I am surprised that the first and the last authors are both from and based in the global north. What was the role of local collaborators in designing and performing this study and analysis? Wouldn't their contribution warrant first or senior (co-)authorship? What was their role in interpreting results and discussing potential benefits, given that they are probably more familiar with the local situation? |  | We appreciate this comment. We followed authorship criteria from ICMJE (International Committee of Medical Journal Editors).  This specific study was nested in the larger Maputo Sanitation (MapSan) Trial, which generated data for our primary and secondary aims, as well as many other analyses. This study was not a primary or secondary outcome, but was described in our study protocol plan. See doi: [10.1136/bmjopen-2022-067941](https://doi.org/10.1136/bmjopen-2022-067941)  Members of the Mozambican team and the US based team have opportunities to lead first author manuscripts using the data generated. Analyses are ongoing. |
| 3. The introduction states that wastewater surveillance emerged only after the emergence of COVID-19. However, it was already commonplace for specific diseases (e.g. polio) or specific places. | We revised the first sentence of the introduction. | We agree that wastewater surveillance was used prior to COVID-19. |
| 4. The introduction states some of the use cases of wastewater surveillance in sewered systems, that include estimate of burden, infection trends, spatial and temporal transmission patterns. It then benefits may be greatest in LMICs. However, all the above use cases depend on a piped and centralized system with few sampling locations that are representative of entire neighborhood and city populations and allow for space- and time resolution, which can not easily be achieved in LMIC settings depending decentralized and often informal wastewater and sewage disposal. It would be much preferrable to state potential use cases that actually are realistic in typical LMIC settings. | We acknowledge the use cases differ on lines 54-56  LMIC settings present challenges to emerging consensus methods for wastewater surveillance and data interpretation, representing use cases that differ in important ways from those in high-income countries.^3^  We further describe constraints to the use cases on lines 69-71:  Use cases may be similar, but would be constrained by poorly defined catchment areas, low sewer coverage, and unclear spatial and temporal representativeness of specific matrices (e.g., open drains, fecal sludge from treatment plants).  We revised the framing of use cases.  We envision a wide range of use cases for ES in LMICs, including some of those now used in WBE: identification of novel or emerging pathogens, monitoring infection trends, and targeting public health response. | We agree there are challenges to environmental surveillance in LMICs. Many of these challenges will be site specific, given |
| 5. The presented methods are inherently biased towards sampling parts of the city with more formal wastewater or sewage collection, which will be neighborhoods that tend to have a better socioeconomical level. The more deprived a subpopulation, the more there will be a tendency to rely on informal disposal mechanisms or seal septic pits and dig new latrines. On the other hand, in the populations the most at risk of infectious diseases will often live in these neighborhoods and will thus be underrepresented in the environmental surveillance approach presented here. This is a very important limitation that needs to be discussed in depth. | We acknowledge the nuance needed for environmental surveillance in the discussion section and have moved this discussion to a stand along paragraph and included additional nuance.  Line 482  However, the utility of fecal sludge as a surveillance matrix depends strongly on the structure of the local fecal sludge disposal chain. In many high-density informal settlements, formal pit emptying is infrequent and sludge is instead buried onsite or removed manually by informal emptiers.^67^ In such contexts, the fecal sludge arriving at a treatment facility may disproportionately represent higher-income neighborhoods, reducing its representativeness for the broader population and limiting its applicability for surveillance. As the burden of disease is often highest in the communities least likely to have their onsite sanitation systems emptied and transported to a treatment plant, methods would be needed to ensure these areas are included in surveillance systems.^67^ Potential approaches include direct sampling of fecal sludge from onsite sanitation systems^9,10^, or sampling coprophagous flies which feed on fecal waste in these systems.^68^ | We agree there is nuance to how these methods would be implemented in low-resource settings. |
| 6. Sample size calculation: 0.5 log10 is a factor of about 3.16, which is a very substantial reduction in concentration, on average over a large city. It would be useful to have sample sizes for more realistic reductions. |  | We acknowledge that these are examples of sample size calculations. Specific sample size calculations would depend on the specific intervention. Our effect size is plausible for specific interventions, such as the impact of improvements to a wastewater treatment plant on effluent and downstream surface water quality. |
| 7. The comparison detection rates of pathogens in this study vs clinical prevalence studies in table S5 shows high discordance between the two methods. This is a very important result given that the authors state public health benefits. This result should thus not be in the supplement but in the main text, and potential reasons for discordance and biases of this study with respect to population prevalence and clinical surveillance should be discussed in detail. |  | We agree this is an important result that requires substantial discussion. We are planning a separate manuscript for this analysis. |
| 8. The above probably highlights that the methods applied here have a problem of representativity of the underlying population if not applied with a much denser spatial and temporal sampling, which is very challenging in terms of logistics and thus has important feasibility and scalability constraints that should be discussed. |  | We agree this is an important result that requires substantial discussion. We are planning a separate manuscript for this analysis. |
| 9. The small number of samples (96 for the entire Maputo in 2 years!) further shows how limited the representativity of this study is and how challenging it would be to extract information on the underlying population level prevalence in space and time beyond simple presence-absence results. |  | We agree there are limitations to the methods that we used. We sampled for approximately six weeks in one rainy season and six weeks in one dry season in one year. We discuss the limitations of our approach in the discussion section on Line 570. |
| 10. Based on the above limitations the claimed potential use cases (introduction, discussion, table S6) need to be much more thoroughly and critically assessed for feasibility, scalability and if they are preferrable to clinical surveillance. |  | We agree careful consideration of the use cases would be needed to tailor any environmental sampling program to the local setting. We are working on other manuscripts that explore potential use cases in detail. |
| 1. The definition of stormwater being only from large standing pools seems peculiar. |  | We were interested testing this matrix and these standing pools existed in the low-income informal settlements in Maputo during the rainy season. |
| 2. Explain unit MPN | We revised line 281 | Agreed |
| 3. Effect of precipitation most likely affects differentially affects different matrices. The authors don't show any results on this. | This information is included in Table S4. | We agree this is an important consideration. |
| 4. Figure 3: I don't think it's appropriate to visualize point < LOD randomly between 0 and LOD. | We revised Figure 2 and 3 to clearly indicate points below the line are non-detects. | Our aim here is to transparently represent the sample size. If we were to exclude these points, then we would only be visualizing a subset of the data. |
| 5. The authors discuss polio surveillance in wastewater. One important difference here is that for polio surveillance, given the disease is almost eradicated, presence/absence information is sufficient. For more common pathogens representative quantification by population is required to infer relevant public health information. |  | We agree methods and data interpretation will depend on the specific use case. |
| 6. LL 500: But at very different concentrations, and because of overflow of combined sewer systems during intense precipitation. |  | Can you please clarify what you mean by “LL 500”? |

Reviewer 4

| 1. Lines 224- 229. Using terms like “pooled regression” and “weighting schemes” can create some confusion in an audience who is not familiar with Bayesian mixed effects regression. Why don’t the authors simply say that they are using a battery of mixed effects models where intercepts and slopes vary across targets? | We revised the methods section and simplified our methods description to avoid confusion. “Pooled” has been removed and substituted with “multi-target” or multi-pathogen” where appropriate. | We appreciate this comment. |
| --- | --- | --- |
| 2. I guess that the analysis assumes that the log-transformed data are normally distributed. The authors should provide some empitical evidence that this assumption is fulfilled. | Empirical data is included in Figure 2 and Figure 3. | Figure 2 and 3 demonstrate that the data was normally distributed within each matrix. |
| 3. Statistical reporting (see the guidelines on the journal web site). Papers that rely on Bayesian methods must include details about the choice of priors and how they were selected. Markov chain Monte Carlo settings should also be reported. | We included this information in Text S2. | Agreed |
| 4. The R code used for the analysis should be included in the supplementary material. This would help the reader to replicate the analysis. | We included this information in Text S2. | Agreed |
| 1. Figures 2 and 3. Dots below the detection limit should be marked differently (use either a different color or symbol). It should be immediately visible that these points are imputed and not observed. | We revised the figure to separately visualize detects and non-detects. | Agreed. |
| 2. Table 2. Credible intervals where both the lower and the upper limit share the same sign should be marked in a special way (e.g. boldface), to help the reader detect significant differences. | Revised Table 2. | Agreed. |
| 3. The statistical analysis is Bayesian. The term “confidence interval” should be replaced by “credible interval” | Revised | Agreed. |
